# Effectiveness of structured self-evaluation of video recorded performance on peripheral intravenous catheter insertion: a randomised control trial study protocol

**DOI:** 10.1186/s13063-023-07200-8

**Published:** 2023-03-11

**Authors:** Orlaith Hernon, Edel McSharry, Andrew Simpkin, Iain MacLaren, Peter J. Carr

**Affiliations:** 1 School of Nursing and Midwifery, University of Galway, Galway, Ireland; 2 School of Nursing, Health Sciences and Disability Studies, St. Angela’s College, Sligo, Ireland; 3 School of Mathematical and Statistical Sciences, University of Galway, Galway, Ireland; 4 Centre for Excellence in Learning and Teaching, University of Galway, Galway, Ireland

**Keywords:** Nursing education, Simulation, Peripheral intravenous catheter training

## Abstract

**Background:**

Clinical psychomotor skills training is a core component of undergraduate nursing education. Performing technical skills competently involves the use of cognitive and motor function. The training of these technical skills is typically carried out in clinical simulation laboratories. Peripheral intravenous catheter/cannula insertion is an example of a technical skill. It is the most common invasive procedure performed in the healthcare environment. Owing to unacceptable clinical risk and complications to patients, it is imperative that practitioners performing these skills are trained effectively to provide patients with best practice and high-quality care. Technologies identified as innovative teaching methods to help train students in this skill and in the skill of venepuncture include virtual reality, hypermedia and simulators. However, little high-quality evidence exists to confirm such educational approaches are effective.

**Methods/design:**

This study is a single-centre, non-blinded, two-group, pre-test and post-test randomised control trial. The randomised control trial will investigate whether a formal structured self-evaluation of videoed performance (experimental group) has an impact on nursing students’ peripheral intravenous cannulation knowledge, performance and confidence. The control group will also be videoed performing the skill but they will not view or self-evaluate their videoed performance. The peripheral intravenous cannulation procedures will be carried out in a clinical simulation laboratory using a task trainer. The data collection tools will be completed online using survey forms. Students will be randomised into the experimental group or into the control group using simple random sampling. The primary outcome measures the nursing students’ knowledge level of the skill of peripheral intravenous cannulation insertion. Secondary outcomes evaluate procedural competence and self-reported confidence and practices in the clinical environment.

**Discussion:**

This randomised control trial will investigate whether this pedagogical approach, using video modelling and self-evaluation, will positively influence students’ knowledge, confidence and performance in the skill of peripheral intravenous cannulation. Evaluating such teaching strategies using stringent methodologies may be impactful in influencing the training provided to healthcare practitioners.

**Trial registration:**

The randomised control trial detailed in this article is an educational research study and so does not fall under the ICMJE definition of a clinical trial as “any research project that prospectively assigns people or a group of people to an intervention, with or without concurrent comparison or control groups, to study the relationship between a health-related intervention and a health outcome”.

**Supplementary Information:**

The online version contains supplementary material available at 10.1186/s13063-023-07200-8.

## Background

Learning clinical psychomotor skills is an integral part of student learning [1, 2]. Performing procedural skills competently involves the student to use cognitive and motor functions. Nurse educators are challenged to deliver student-centred active learning strategies, to prepare students for practice. In doing so educators effectively incorporate cognitive skills with technical skills with the intention to boost self-confidence and efficacy in those skills [3]. They are challenged to prepare nursing students to provide safe and competent care, as it is noted that the care a patient receives is linked to the competence and performance of nurses [4]. In nursing education these technical skills are taught in clinical laboratory settings. This setting is the foundation of safe patient care [5] and the benefits of clinical skills laboratories are well documented and are integral in the teaching of procedural skills [6].

The insertion and management of a peripheral intravenous catheter/cannula (PIVC) is an important nursing task [7]. They are the most commonly used invasive device and up to 70% of patients need a PIVC during their hospitalisation [8]. The procedural task is performed in order to provide patients with intravenous fluids and/or medication [9]. Although PIVCs are a globally common invasive procedure their premature failure, leading to their removal and replacement is also very common [10, 11]. The insertion, management and removal of a PIVC can have negative experiences for patients and there is a need for the procedure to be improved and to adhere with recommended clinical guidelines [11]. However, there are inconsistencies between recommended practice and reported practice [10]; Cooke et al. highlight that in order to provide best practice and quality care, it is paramount to produce effectively trained practitioners [11]. Nurses should receive appropriate training incorporating knowledge and application of psychomotor skills and thereafter be supervised in their practice in order to become competent in intravenous cannulation [7]. Currently, Irish PIVC training standards include a hybrid learning model. The programme includes both the theoretical and practical components [12]. Published as a national training standard it was recently expanded to include the undergraduate nursing and midwifery curriculum [13]. The practical component of PIVC insertion includes an observation and demonstration session in a clinical skill laboratory/simulation environment [12].

Recent studies regarding the teaching of PIVC and venepuncture have been noted to use innovative teaching methods such as web-based learning or tools [1, 14, 15], hypermedia [16], simulators [5, 9, 17, 18], video-assisted teaching [18], virtual reality [19], video-assisted debriefing [20] and a vein visualisation display system using near-infrared light [21]. An integrative review regarding the teaching of venepuncture similarly identified technologies such as e-learning courses, videos and demonstrations combined with manikins or haptic devices [22].

This proposed study will determine if video technology can assist with improvements in the teaching and assessment of PIVC insertion in an undergraduate nursing student cohort.

As a teaching method, video modelling is the use of video recordings which target particular skills and it allows students to view their own performance. The intention is to increase learning by evaluating areas which need improvements or adjustments [23]. One of the goals of this proposed study is to use a teaching strategy which can promote student-centred, active learning to improve the student’s competency in the clinical skill of intravenous cannulation. Combining video recording and reflection may be an effective teaching strategy for learning clinical skills [23]. Studies using quasi-experimental, surveys and descriptive and interpretive designs report the beneficial use of video modelling to teach clinical skills. They include the development of self-evaluation, reflection and skill performance [24]. It can assist in promoting the student’s self-awareness [23, 25], which is noted to improve clinical skills competency [25]. Students reviewing and reflecting on their performance are playing an active role in their learning. As a result, their clinical skill competency can be enhanced, and it can promote self-directed learning.

A more detailed overview of such studies includes first year midwifery students self-recorded and assessed their videos of selected midwifery practice skills. The study found that this provided an accessible option for assessing competency, self-reflection and re-recording to master their skill [26]. Comparing the effect role-playing and video feedback has on learning cardiopulmonary resuscitation, the study noted that the mean difference between the psychomotor and cognitive learning scores before and after training in the video self-feedback group was significantly higher [27]. Another study focused on the skills of intramuscular injections and suction and evaluated the effectiveness of using video recording and self-evaluation [28]. The experimental group, who self-practised, video-recorded and self-evaluated the skill had statistically significantly better performance in nursing skills and confidence in comparison to the traditional group, of lecture and demonstration. Similarly, Cernusca, Thompson and Riggins (2018) identified that the use of iPad videos appears to be an effective teaching tool, regarding learning sterile procedures, for students to assess their performance and reflect on areas which need development [23]. A study on self-evaluation of performance videos, which focused on urinary catheterisation, noted that self-evaluation of performance videos was effective and students in the experimental group demonstrated higher competency in the skill, their communication skills and their motivation to learn. It appeared to positively influence students’ retention of knowledge as they actively participated in their learning [25].

Previous study findings give validation for conducting this research study. A recent systematic review on the effect of video on psychomotor skills in nursing noted that video would be a good addition to teaching and learning clinical skills [2]. Although the use of video is not novel, it does not appear to have been studied in the context described in our study. Corroborating our stance that our proposed study was not carried out previously on this topic, a systematic review [2] did not appear to identify a randomised control trial study whereby students reviewed their own intravenous cannulation videos. Creating a safe and controlled environment for students in order for them to learn and practice their psychomotor skills, which are required to become a competent practitioner, is the responsibility of nursing programmes [25].

The aim of our study is to evaluate the effectiveness of structured self-evaluation of video-recorded simulation practice on nursing students’ PIVC knowledge, confidence and performance. Outcome measures will focus on procedural knowledge, procedural competency and students’ confidence in the skill. Alongside clinical outcomes regarding students’ successful and unsuccessful attempts and feedback on their performance of the skill will be investigated in this study. Our study is designed to assess the use of video technology in order to provide students with practice opportunities and to promote directed learning which the student can carry out independently. It does not wish to increase the educator burden. The results of the study will assist educators in designing and developing a data-informed curriculum on PIVC care and insertion.

## Objectives

### Research hypothesis

Formal structured self-evaluation of videoed performance has a positive impact on nursing students’ peripheral intravenous cannulation knowledge, performance and confidence.

### Study objectives

The primary objective of this study is to compare the effect of structured self-evaluation of video-recorded performance on students’ knowledge of peripheral intravenous cannulation to students who do not formally self-evaluate their videoed performance.

Secondary objectives include comparing the effect of structured self-evaluation of video-recorded performance on students’ skill performance, their confidence levels and the success of their attempts in clinical practice to students who do not formally self-evaluate their videoed performance.

## Methods

### Study design

The study protocol has been prepared and is reported in accordance with the SPIRIT PRO extension reporting guidelines [29]. This study protocol describes a single-centre, non-blinded, two-group, pre-test and post-test randomised control trial. It is not possible to blind students to their randomisation due to the nature of the study. The study design is open label with only outcome assessors being blinded.

### Study setting and participants

The study interventions and data collection will be performed online and in a simulated clinical skills laboratory. The student group will be final year nursing students registered in a nursing programme in one Irish university.

### Eligibility criteria

Students will be asked to consent onto the study by signing an informed consent form prior to participating in any study interventions/data collection. The informed consent will be taken by the primary researcher with the support of the wider research team. On the consent form, students are asked to consent to the study results being shared with the affiliated School of Nursing and Midwifery, and training hospitals and that results may be published. This trial does not involve collecting biological specimens for storage. Study eligibility criteria can be noted in Table 1.

**Table 1:** Study eligibility criteria

**Inclusion criteria**
Final year nursing students in the participating university
Final year nursing students who have completed the formal training required on the skill as per national curriculum standard requirements
**Exclusion criteria**
Nursing students from other years will not be included nor will nursing students in postgraduate courses
Midwifery students will not be included
Inability to commit to study procedures/interventions

### Intervention groups

Students will be on clinical placement throughout this study period. During this time period, they may get opportunities to perform the skill under supervision in clinical practice. This study will include two groups. Students will be randomised into an experimental group or a control group. The study comparator between both groups is that the experimental group will view and self-evaluate their recorded performance using a task-specific checklist. Enrolled participants will be randomised in equal proportions, where possible, between the experimental group and the control group. Participants will receive a regular email to remind them of study interventions/data collection procedures. Participants will be given a study number which they will be asked to input at each data collection point. This will allow the research team to follow up with participants and link in if study interventions/data collection is not performed. There is no anticipated harm and compensation for trial participation, therefore there are no provisions considered necessary for post-trial care.

#### Experimental group

The experimental group will receive current education standards however they will have two more simulated practices. These extra practice sessions will be video recorded. After completing their extra practice students will provide feedback to the researcher on how they perceived the practice session. Students will then get the opportunity to view their video-recording and complete a self-evaluation checklist and update the researcher on any feedback they may have after viewing their performance and completing the task-specific checklist. The performance will be evaluated by the researcher. This process will be repeated for the students’ second practice session a number of weeks later. Students in this group will complete a knowledge, attitude and practices (KAP) survey prior to their first practice session (pre-test) and after their second practice session (post-test). Students will complete a clinical outcomes survey, evaluating their practices in the clinical environment, throughout the study.

#### Control group

The control group will receive current education standards however they will have two more simulated practice sessions. Similar to the experimental group these practice sessions will be video recorded and after completing their extra practice students will provide feedback to the researcher on how they perceived the practice session. Students will not get the opportunity to view their video and self-evaluate. The students’ performance will be evaluated by the researcher. This process will be repeated for the students’ second practice session a number of weeks later. Students in this group will complete a KAP survey prior to their first practice session (pre-test) and after their second practice session (post-test). Students will complete a clinical outcomes survey, evaluating their practices in the clinical environment, throughout the study.

#### Criteria for discontinuing or modifying allocated interventions

The study interventions for both experimental and control groups will be discontinued at the participants’ request. There will be no special criteria for modifying allocated interventions.

### Study outcomes

#### Primary outcome

The primary outcome in this study is the students’ knowledge level of the skill of PIVC insertion.

#### Secondary outcomes

Secondary outcomes include the students’ procedural competence, confidence and practices in the clinical environment as well as their feedback of their performance of the skill and of the intervention. All outcomes will be measured using data collection tools; KAP survey, task-specific checklist, feedback sheet and clinical outcomes survey.

### Participant timeline

**Fig. 1:**
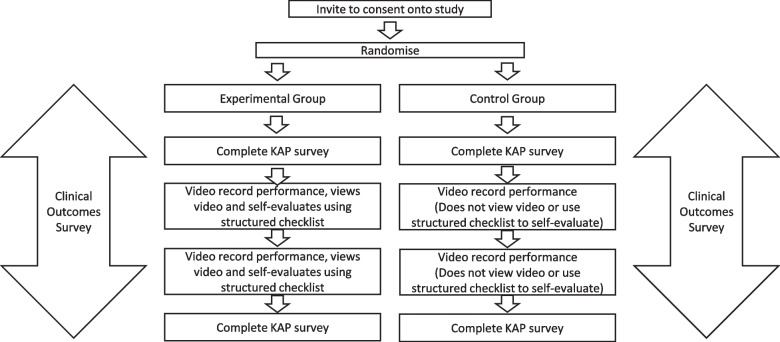
Study design

This study is envisioned to take 5–6 months. The study will start when the participants commence their internship placement in January 2023 and will end once all study interventions and data collection have been completed. The study design can be noted in Fig. 1.

### Sample size

A sample size calculation was carried out by a statistician to identify the number of students required in each group to adequately power this study. A previous study found students achieved an average score of 7.2 out of 15 in a KAP survey in relation to venepuncture and peripheral intravenous cannulation (SD = 2.4) [30]. A sample size of *n* = 46 students (i.e., 23 in each group) is required to find a 25% increased score in the intervention group (i.e., an average score of 9). This was calculated for 80% power with an alpha of 0.05 under a one-sided hypothesis for a two-sample *t*-test. To account for attrition, we will invite all students (*n* = 69) in the class to participate in the study. Based on the experience of student nursing studies, we expect between 10 and 30% attrition. Given the total *n* = 69 student recruitment, we therefore expect that statistical power of 80% will be maintained after attrition.

### Recruitment

Students will be recruited onto the study by invitation, both in writing and face-to-face. Every student in the class will be invited to consent during scheduled university time. As students are to be consented prior to the randomisation and to ensure even numbers in both control and experimental groups, students will need to decide on their participation by a certain date, before study interventions and data collection are due to start.

### Randomisation

#### Sequence generation

Participants will be randomly assigned to either the control or experimental group using simple randomisation. Once consented each participant’s study number will be inserted into an Excel document. Once every participating student has consented and is inputted into the Excel sheet, we will create a set of uniform random numbers, one for each participant. The random numbers will then be sorted from lowest to highest. The first half of the group will be in the intervention group and the second half of the group will be in the control group. Once the participants have been randomised, students will be made aware of their intervention group. In the case of an odd number of consented participants, the experimental group will have the higher number.

#### Concealment mechanism

The study number allocated to participants will be assigned as they complete their consent form however the randomisation will not be complete until all participating students have signed their consent. The uniform random numbers will be created using the random function in Excel; therefore, the group in which the students will be assigned to is concealed, until random numbers are assigned and then sorted from lowest to highest.

#### Implementation

The primary researcher will implement the randomisation process. As the researcher will not be involved in teaching the students and will not be familiar with the students, this will reduce the risk of bias. All students will be allocated to their randomised group at the same time, students cannot be randomised at the time of consent as the research team will be unaware of the overall study sample size. Therefore, to ensure equal allocation as best as possible, randomisation will not be completed until every participant has consented. The allocation list will be kept electronically and only available to the research team. However, the allocation details of each participant are not confidential as students will know their allocated group.

### Data collection methods

#### KAP survey

Both control and experimental groups will complete a KAP survey pre-intervention and post-intervention. This will be disseminated to students using an online survey platform. The KAP survey consists of knowledge, attitude and practice questions using both closed and open-ended questions. The survey was initially designed to evaluate students on intravenous cannulation and venepuncture skills. The survey underwent face and content validity by vascular access experts/educators and public and patient involvement members. For this study, only questions pertaining to the intravenous cannulation procedure will be collated. Students from both groups will complete the same KAP survey.

#### Feedback

Students will be asked to provide feedback regarding their simulation experience. Prompts may be used to help direct students to provide feedback. However, the goal will be to ascertain how they felt regarding the experience, what they think should be done differently to improve the experience and how they plan to incorporate these improvements into their next experience whether simulated or clinical.

#### Task-specific checklist

A task-specific checklist which was developed from the procedural task as recommended by the national guidelines [12] will be used by both the experimental group and the researchers. When students in the experimental group are self-evaluating their practice, they will be guided by this checklist where they will decipher whether their clinical performance followed the recommended guidelines. The steps where relevant will be embedded with knowledge criteria, such as the concentration of the skin preparation wipe or whether asepsis was maintained. This is to provide students with more than just a step-by-step checklist but to embed both knowledge and procedural skill and to provide them with a data-informed step-by-step checklist. Researchers when evaluating each student’s videoed performance will use this checklist. This will allow researchers to compare within groups and between groups. It will allow researchers to investigate if students’ evaluation reports are similar to the evaluation completed by the researchers.

#### Clinical outcomes survey

Both student groups will be asked to complete the clinical outcomes survey throughout the study period. This survey will capture self-reported data on students’ success rates and practices regarding the skill in clinical practice.

### Retention

Once a student is enrolled and then randomised, the study team will make every reasonable effort to follow the student for the complete study period. Providing all participating students with extra practice opportunities that they would not get during the standard curriculum may help retain participants and promote follow-up. Regular communications regarding data collection may promote follow-up. However, participants may withdraw from the study for any reason up to the time of data analysis without any negative consequences.

### Data management

Data entry will be completed using survey forms which will be housed online, using a third-party platform. Students will be identified and followed throughout the study period using their own study numbers. Students will be provided with their study number after they have consented and will be given a copy of their number to input into each of the data capture tools. All data (electronic and paper) pertaining to students will be stored securely with access only available to the research team. There is no data monitoring committee as this is considered a low-risk intervention. An end-of-study report will be provided to the ethics committees.

### Data analysis

Study data will be analysed to explore the impact self-evaluation of videoed performance has on nursing students’ knowledge, confidence and performance. Data will be inputted electronically using online survey forms, this data can then be exported for analysis. For qualitative survey data analysis, data obtained from the open-ended questions and feedback, coding will be used. The codes chosen will be decided on as they emerge from the data. The quantitative data gathered will be first analysed using descriptive statistics and graphical summaries. To investigate the efficacy of the intervention, an analysis of covariance (ANCOVA) model will be used with the follow-up KAP score as the response, group as the key exposure (control vs intervention) and baseline KAP score controlled for as a covariate. Missing data will be reported on and where appropriate multiple imputation will be used for analysis. Intention-to-treat analysis will be used, and participants will be analysed using the groups they were randomised to. No interim analysis will be performed as this is a short-term study and the final knowledge outcomes are only assessed at the end of the study. Given the intervention and study end points, we do not expect adverse events and to be required to stop the study. The datasets analysed during the current study and statistical code are available from the corresponding author on reasonable request, as is the full protocol.

## Discussion

Prior feedback from past students noted that they wanted more practice opportunities [30] Therefore, it was considered appropriate to ensure all students received extra simulated practice. Ensuring every participating student receives extra practice sessions allows us to more closely evaluate whether the intervention of formal self-evaluation is effective. Simulation and adult education are examples of teaching strategies which nursing schools use in their programmes [31]. It is widely accepted that nurse educators recognise and use appropriate teaching strategies in order to meet the requirement of the curriculum and the learning needs of students [25]. This study consists of video technology, self-evaluation and simulation. The intervention proposed in this study incorporates directed learning, active learning and technology. Students will be required to reflect back on their performances of intravenous cannulation and identify what skills need further refinement. Education theorists suggest this reflection on performance activates the students’ metacognition and increases their control of their own learning [32]. There is a need to evaluate such teaching methods using rigid research methodologies in order to create evidence-based, data-informed curriculums.

Assessment is another core element in teaching and learning. Evaluating the clinical skills performed by students is a challenge for nurse educators alongside creating innovative teaching methods while providing an educational experience that will contribute to safe nursing care. It is important to have tools and strategies available to help us demonstrate the learning and progression of clinical skills [15]. Therefore, it is important to design and evaluate the effectiveness of innovative assessment strategies, such as video and self-evaluation proposed here. Trialling different teaching and assessment strategies may be the only way to distinguish which method results in the best outcomes. Previous studies have noted the requirement for more stringent methodologies, such as randomised control trials [7, 28].

## Conclusion

Education and clinical instruction can lead to better PIVC outcomes. However, it is important to assess the effectiveness of educational technologies and associated teaching strategies for this common invasive procedure. This study represents a randomised control trial to investigate if self-evaluating videoed peripheral intravenous cannulation performance has an effect on students’ knowledge, confidence and clinical performance for PIVC insertion.

### Trial status

Recruitment commenced in November 2022 and data collection will begin in January 2023. This is protocol version 1.0, 19–12-2022.

## Supplementary Information

**Additional file 1.** 
